# Taking the steps toward sustainable livestock: our multidisciplinary global farm platform journey

**DOI:** 10.1093/af/vfab048

**Published:** 2021-10-20

**Authors:** M Jordana Rivero, Alex C O Evans, Alexandre Berndt, Andrew Cartmill, Andrew Dowsey, Anne Farruggia, Catherine Mignolet, Daniel Enriquez-Hidalgo, Dave Chadwick, Davy I McCracken, Dennis Busch, Fabiana Pereyra, Graeme B Martin, Gregg R Sanford, Helen Sheridan, Iain Wright, Laurent Brunet, Mark C Eisler, Nicolas Lopez-Villalobos, Pablo Rovira, Paul Harris, Paul Murphy, A Prysor Williams, Randall D Jackson, Rui Machado, Suraj P.T., Thomas Puech, Tommy M Boland, Walter Ayala, Michael R F Lee

**Affiliations:** 1 Sustainable Agriculture Sciences, Rothamsted Research, North Wyke, Okehampton, Devon, UK; 2 School of Agriculture & Food Science, University College Dublin, Belfield, Dublin 4, Ireland; 3 Embrapa Southeast Livestock, São Carlos, São Paulo, Brazil; 4 School of Agriculture, University of Wisconsin–Platteville, Platteville, WI, USA; 5 Bristol Veterinary School, University of Bristol, Langford, Somerset, UK; 6 *INRAE—ACT UE 0057 DSLP, 17450 Saint Laurent de la Prée, France; 7 INRAE—ACT, UR 0055 ASTER, Mirecourt, France; 8 School of Natural Sciences, Bangor University, Bangor, UK; 9 Hill & Mountain Research Centre, SRUC: Scotland’s Rural College, Kirkton Farm, Crianlarich, UK; 10 Instituto Nacional de Investigación Agropecuaria (INIA), Treinta y Tres, Uruguay; 11 UWA Institute of Agriculture, The University of Western Australia, Crawley, Australia; 12 **Department of Agronomy, University of Wisconsin–Madison, Madison, WI, USA; 13 International Livestock Research Institute (ILRI), Nairobi, Kenya; 14 School of Agriculture and Environment, Massey University, Palmerston North, New Zealand; 15 Livestock Research Station Thiruvazamk unnu, Kerala Veterinary and Animal Sciences University, Kerala, India; 16 Harper Adams University, Newport, Shropshire, UK

**Keywords:** circularity, grazing systems, mixed farming, precision farming, research farms, ruminant livestock

**Implications**

- The Global Farm Platform was conceived and established to explore multidisciplinary strategies for optimising the sustainability of ruminant livestock systems around the world.
- International sustainability issues are common, but the solutions are often region-specific; therefore, our farms, situated across all major agroclimatic zones, are a unique resource worldwide.
- Each farm is following ‘steps to sustainable livestock’ to improve their production system(s), thereby developing robust metrics to progress economic, environmental and social viability.
- The consortium works collaboratively to improve the sustainability of ruminants, which we argue are a vital component of global food systems, delivering both human and planetary health.

Ruminant livestock are a vital global source of high-quality protein and bioavailable minerals and vitamins. They support healthy dietary choices by providing milk and meat produced from less productive land and food industry byproducts. However, despite the contribution of ruminants to food systems and the circular bioeconomy, ruminant production systems are increasingly questioned due to their environmental impact, particularly their significant contribution to greenhouse gas (**GHG**) emissions and associated global warming. There is a need, therefore, to identify a pathway to sustainable global ruminant production. In 2014, our group defined eight strategies or “steps” (Eisler et al., 2014), to mitigate the environmental impacts of ruminant production while optimizing the quantity and quality of the food they produce. To realize these goals, we established the “Global Farm Platform” initiative (www.globalfarmplatform.org), a network of “farm platforms” or research farms (RFs), to explore multidisciplinary strategies and evaluate different production systems around the globe (Table 1). Here, we provide a perspective on our approach and the steps we are taking to realize the ambition of supporting sustainable ruminant livestock production as a part of future food systems contributing to both human and planetary health.

**Table 1.** Global Farm Platform steps toward sustainable livestock systems in our network of 16 RFs

	Steps to sustainable livestock (see Eisler et al., 2014)							
Research farm (see Rivero et al., 2021)	1	2	3	4	5	6	7	Best Practice (8) and main aim
**Dairy 1** (Palmerston North, New Zealand)	●	●	●	●	●	●	●	Temperate grazing dairy system—improve sustainability, farmer and animal welfare, and profitability through once a day milking and selection of dairy cows for feed conversion efficiency.
**ESL** - Embrapa Southeast Livestock (Sao Carlos, Brazil)	●	●	●	●	●	●	●	Subtropical sustainable beef and milk systems—explore net-zero C potential.
**HAUF** - Harper Adams University Farm (England, UK)	●		●			●	●	Conversion to circular farming—show how mixed- farming can deliver to net-zero C.
**HRC** - Henfaes Research Centre (Wales, UK)	●						●	Temperate uplands sheep—improve productivity with least environmental impact.
**INIA-PAP** - INIA Palo a Pique (Treinta y Tres, Uruguay)	●		●	●	●	●	●	No-till crop-livestock (beef) rotations—evaluate four ways of producing 400 kg LW/ha per yrs.
**INRAE-AM** - INRAE ASTER-Mirecourt (Mirecourt, France)	●	●	●				●	Organic crop-livestock (dairy) system—implement an agroecological transition.
**INRAE-SLP** - INRAE Saint-Laurent-de-la-Prée (La Rochelle, France)	●	●	●	●	●		●	Organic crop-livestock (beef) system in marshes—restore biodiversity, mitigate GHG, produce animal and vegetal human food for short circuit, enable adoption by farmers.
**JOC** - The John Oldacre Centre for Sustainability and Welfare in Dairy Production (England, UK)			●				●	Precision farming system for housed dairy cattle— monitoring animal health, behaviour and welfare, nutrition and GHG emissions.
**KRS** - Kapiti Research Station and Wildlife Conservancy (Nairobi, Kenya)	●	●	●				●	Semi-arid rangeland (livestock-wildlife)—improve livestock production sustainably, explore the ecological dynamics of savannahs and their interactions with humans, livestock and wildlife
**NWFP** - North Wyke Farm Platform (England, UK)	●	●	●	●	●		●	Temperate lowland sheep and beef systems—assess sustainability of production systems in its three dimensions.
**UWP-PF** - University of Wisconsin- Platteville Pioneer Farm (Wisconsin, US)	●			●		●	●	Dairy (housed or hybrid grazed systems)—investigate the effects of alternative dairy production systems on water quality and nutrient cycling.
**SRUC-KA** - SRUC Kirkton and Auchtertyre (Scotland, UK)	●	●	●		●	●	●	Temperate uplands livestock (sheep and beef)—understanding what may be practical or economically viable for upland land managers to implement to improve sustainability.
**SVT** - Silent Valley Thiruvazhamkunnu Livestock Research Station (Kerala, India)	●	●	●	●			●	Cut and carry livestock systems—assess sustainability of different fodder management strategies.
**UCD-LTGP** - UCD Lyons Farm Long Term Grazing Platform (Dublin, Ireland)	●		●		●		●	Temperate lowland dairy x beef systems—investigate the interrelationships between pasture type, animal production, the environment, product quality and farm economics.
**UWA-FF** - University of Western Australia Future Farm 2050 (Pingelly, Australia)	●	●	●		●		●	Drylands sheep system—define and implement the ‘ideal’ farm: profitable, ‘clean, green and ethical’, commitment to conservation of biodiversity; it must take into account the people’s needs.
**WICST** - The Wisconsin Integrate Cropping Systems Trial (Wisconsin, US)	●		●		●	●	●	Midwestern cropping systems - evaluate productivity, profitability, and ecological performance.

● Step being addressed by the research farm (RF) as part of their research activities.

## Feed Animals Less Human Food (Step 1) 

Most of our RFs are investigating ways to enhance the sustainability of forage-based systems, with no use, or only strategic use, of supplementary feeds for certain short periods of the production cycle (Rivero et al., 2021). INRAE-SLP decreased the percentage of arable lands dedicated to production of supplemental feed for animals from 48% in 2017 to 28% in 2020. All ruminants in the INRAE-AM system are fed exclusively on grass, while the annual crops are intended exclusively for human consumption. SVT has introduced a new cultivation strategy, the Kenyan “Tumbukiza” method, which uses cultivars of Hybrid Napier (*Pennisetum purpureum* L. × *Pennisetum glaucam* L.) planted in holes to improve soil fertility and moisture levels, thus increasing fodder biomass production for their cut and carry system. UCD-LTGP and HRC are testing grazing systems based on swards with increasing levels of plant diversity (perennial ryegrass monoculture, perennial ryegrass and white clover mixed sward, and a 6-species grass, legume, forage herb mixed sward) to enhance resilience to extreme weather events and deliver greater yield with reduced inputs.

## Raise Regionally Appropriate Animals (Step 2)

We have identified the need for selecting animals adapted to local conditions that are able to cope with climate change challenges (Rivero et al., 2021). INRAE-AM is adapting its animals to low-input grazing systems (e.g., enhanced rusticity and reduction of cow size) via selection and crossbreeding. INRAE-SLP bases its research on a dual-purpose local rustic beef breed native to wetlands (Maraîchine), while SRUC-KA is crossbreeding Aberdeen Angus with Beef Shorthorn cattle in order to improve their ability to cope with extreme mountainside environments. Similarly, SVT is working with native breeds of cattle (Vechur), buffalo (Murrah), and goats (Malabari and Attapadi) plus *indicus* × *taurus* crossbred cattle, with the former having been shown to exhibit greater tolerance to heat stress (Elayadeth-Meethal et al., 2018).

## Keep Animals Healthy (Step 3)

Most of our RFs are working in this area with different approaches. For instance, the use of sensors and additional technology allows SRUC-KA to monitor animal health and welfare in mountainous conditions, while JOC is using 64 video cameras to track cattle movements and social behavior for early disease detection and to assess infectious disease transmission and minimize antimicrobial resistance. KRS demonstrated that a vaccine against wildebeest-associated malignant catarrhal fever is highly effective against the disease in cattle with a vaccine efficacy of 80% (Cook et al., 2019). Through work at SVT, welfare challenges in subsistence dairy farms in India have been identified (Mullan et al., 2020). UCD-LTGP is showing that greater diversity in forage plants decreases animal parasite burdens.

## Adopt Smart Supplements (Step 4)

**Figure 1.**
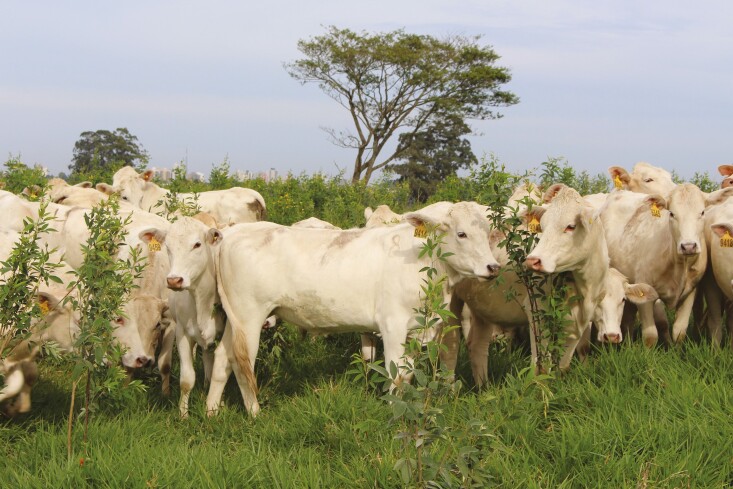
Canchim breed heifers in an *Urocloa brizantha* pasture enriched with *Cajanus cajan* cv. BRS Mandarim legume (Photo: Gisel Rosso).

In some of our RFs, spontaneous vegetation is being explored as feed, bedding (e.g., reed in INRAE-SLP; Durant et al., 2020), or smart supplements (e.g., *Azolla* spp.—a small aquatic fern that flows on the water surface and is nutritionally rich—in SVT and INRAE-SLP). ESL has developed the Guandu BRS Mandarim (*Cajanus cajan* cv. BRS Mandarim), an N-fixating legume suitable to enrich soil quality of degraded pasturelands while its aerial part serves as a protein supplement to cattle, particularly in the dry season (Figure 1). HAUF and UWA-FF are also testing dietary supplements or feed ingredients which act as methane suppressants at a farm system scale.

## Eat Quality Not Quantity (Step 5)

Even though this step is mainly oriented toward the consumer, many of our RFs are working on improving the quality of the final food products. In addition to increasing system productivity, SRUC-KA is focusing on carcass conformation through the use of CT scanning (Lambe et al., 2017), the NWFP is investigating the nutritional value and the associated carbon footprint of forage-based beef systems (Lee et al., 2021), and UCD-LTGP has ongoing research on meat quality from multispecies forage leys.

## Tailor Practices to Local Culture (Step 6)

Most of the researches undertaken by our RFs are agreed with and/or transferred to stakeholders, particularly the farming community. WICST seeks to transform agriculture of the North Central United States to perennial grassland dominance to restore the function of the original prairie—water purification, flood mitigation, climate stabilization, and biodiversity—while revitalizing rural communities decimated by farm consolidation. INIA-PAP is testing four crop-livestock (beef) rotations, representative of the predominant commercial livestock strategies in Uruguay, with the aim of evaluating four ways of producing 400 kg LW/ha per year that is economically, environmentally, and operationally viable (Rovira et al., 2020). UWP-PF is investigating the effects of alternative dairy production systems on water quality and nutrient cycling. Dairy 1 is evaluating breeds and crossbreeding for once-a-day milking (Jiang et al., 2020) and the use of precision technology to feed cows more efficiently (Duranovich et al., 2021). HAUF is mapping the impact (economic, environmental, and social indicators) of conversion from separate crop and livestock enterprises to a mixed circular crop-livestock farming system. HRC has identified the cultural, practical, and economic barriers to better soil and nutrient management in ruminant systems (Gibbons et al., 2014; Rhymes et al., 2021).

## Track Costs and Benefits (Step 7)

All our RFs are delivering to this step with various approaches. HRC found that urine patches deposited on hill and upland soils generate very small quantities of nitrous oxide, with implications for carbon footprinting (Marsden et al., 2018). UCD-LTGP is investigating the impact on above- and below-ground biodiversity, water quality, meat quality, economic, and other non-market benefits of sustainable grazing systems. ESL has demonstrated that crop-livestock and crop-livestock-forest integrated systems deliver less nitrous oxide into the atmosphere as compared with conventional crop practices (Sato et al., 2019). The NWFP is applying Life Cycle Assessment (LCA) approaches to compare its production systems (McAuliffe et al., 2018), while INIA-PAP is collating a database to apply LCA to its four crop-livestock systems.

## Study Best Practice (Step 8)

Our vision is to identify better practices to optimize the use of livestock in various regions, using local resources, breeds, and feedstuffs—and produce tangible evidence of sustainability. The “Global Farm Platform” initiative started with three operational RFs in three continents in 2014 and has subsequently grown to 16 RFs in five continents covering a wide variety of social and agroclimatic conditions and production systems (Table 1). There are plans to continue establishing further platforms to test other relevant ruminant production systems, for example, two Chinese RFs and another Australian RF are in the process of joining.

## Final Remarks

Our network of RFs traverses a wide variety of social and agroclimatic conditions and production systems, and also brings together researchers with expertise in most of the areas relevant to the multidisciplinary approach required to address the global issues contributing to sustainable animal production, such as animal health, welfare, nutrition and genetics, pasture management, agroecology, biodiversity, agroforestry, silvopastoralism, meat quality and safety, GHG emissions, hydrology, soil carbon, biogeochemistry, LCA, economics, knowledge exchange and extension, precision farming and sensors, informatics, statistics, modeling, and artificial intelligence.

Since our first paper on the steps to sustainable livestock was published (Eisler et al., 2014), there has been a major increase in recognition that livestock managers play a vital role in managing land, from the perspectives of carbon sequestration and biodiversity, among other benefits, such as wildfire control (FAO, 2020). Furthermore, the role of farmed livestock in the circular bioeconomy has been recognized (Van Zanten et al., 2019), as has the potential for Precision Livestock Farming, further strengthening the commitment of our RF network to the exploration of solutions needed for the next steps toward sustainable livestock. Despite their variation, our farms face the same challenges–reducing environmental impact, improving animal performance, and maintaining health and welfare–yet, the solutions to these challenges must be regional and applied under local conditions, verifying the value of our network across contrasting agroclimatic zones as a global resource.

Single metrics of sustainability, such as methane intensity/carbon footprint, seem to favor intensive solutions for ruminant production. However, in such solutions, there are tradeoffs in relation to, for example, the food/feed competition and the ability of the animals to express their natural behavior. Our team has acknowledged these tradeoffs as critical issues in choosing the major steps to sustainable livestock production, and we decided to favor forage-based solutions. Forage-based systems are inevitably complicated by the largely uncontrolled environment within which the animals and the forage plants need to survive and thrive. An obvious major limitation is the seasonal nature of rainfall and temperature, but successful responses of these challenges can be found by making visionary choices for both animal genotype and forage species. For example, by moving away from “traditional” forages, we have found species that offer nutritional advantages, drought resistance, shelter for neonates, and plant secondary compounds that combat helminths and methane emissions. Few if any of these alternative forages have been subjected to genetic selection, so there is an opportunity for improvement. Finally, increasing forage diversity, and thus offering dietary diversity, improves animal productivity and health.

## About the Authors

### “Global Farm Platform” Initiative

The Global Farm Platform initiative (www.globalfarmplatform.org) is a network of research farms and institute members working collaboratively to enhance the sustainability of ruminant livestock systems through the development of transformational regional solutions to global challenges and promote their adoption. This multidisciplinary international network will provide a unique combination of research and practice for diverse ruminant production systems in a wide range of cultural, socioeconomic, and climatic zones.

Research farms:

- **Dairy 1**, Massey University, Palmerston North, New Zealand
- Embrapa Southeast Livestock, Embrapa, Sao Carlos, Brazil (**ESL**)
- Harper Adams University Farm, Harper Adams University, England, UK (**HAUF**)
- Henfaes Research Centre, Bangor University, Wales, UK (**HRC**)
- INIA Palo a Pique, INIA, Treinta y Tres, Uruguay (**INIA-PAP**)
- INRAE ASTER-Mirecourt, INRAE, Mirecourt, France (**INRAE-AM**)
- INRAE Saint-Laurent-de-la-Prée, INRAE, La Rochelle, France (**INRAE-SLP**)
- The John Oldacre Centre for Sustainability and Welfare in Dairy Production, University of Bristol, England, UK (**JOC**)
- Kapiti Research Station and Wildlife Conservancy, ILRI, Nairobi, Kenya (**KRS**)
- The North Wyke Farm Platform, Rothamsted Research, England, UK (**NWFP**)
- University of Wisconsin-Platteville Pioneer Farm, Wisconsin, US (**UWP-PF**)
- SRUC Kirkton & Auchtertyre, Scotland, UK (**SRUC-KA**)
- Silent Valley Thiruvazhamkunnu Livestock Research Station, KVASU, Kerala, India (**SV**)
- UCD Lyons Farm Long-Term Grazing Platform, Dublin, Ireland (**UCD-LGP**)
- University of Western Australia Future Farm 2050, Pingelly, Australia (**UWA-FF2050**)
- The Wisconsin Integrate Cropping Systems Trial, University of Wisconsin-Madison, Wisconsin, US (**WICST**)

In addition to the institutions hosting the research farms, there are other institute members that contribute to the network:

- Key Laboratory of Plant Resources, Chinese Academy of Science, China (**CAS**)
- International Centre for Tropical Agriculture, Kenya (**CIAT-Kenya**)
- China Agricultural University, China (**CAU**)
- Federal University of Agriculture, Abeokuta, Nigeria (**FUNAAB**)
- Department of Agronomy, Kansas State University, US (**KSU**)
- Small Scale Livestock and Livelihoods Program, Malawi (**SSLLP**)
- Soil Science Department, College of Agriculture and Natural Sciences, University of Cape Coast, Ghana (**UCC**)
- Faculty of Agricultural, Life and Environmental Sciences, University of Alberta, Canada (**UoA**)
- Institute of Dairy Science, Zhejiang University, China (**ZU**)

World map with the location of the Research Farms and Member Institutes that are part of the Global Farm Platform initiative
